# Coincidental occurrence of endometrial hyperplasia with atypia and vaginal leech infestation in a postmenopausal woman: a rare case report

**DOI:** 10.1097/RC9.0000000000000348

**Published:** 2026-03-09

**Authors:** Musie Negasi Gebreslase, Birhanu Kassie Reta, Henock Solomon Sisay, Haftom Guesh Girmay, Kibrom Tsegay Hailu, Selamawit Muluken Alemu

**Affiliations:** a Department of Obstetrics and Gynecology, Aksum University College of Health Science, Aksum, Ethiopia; b Department of Pathology, Aksum University College of Health Science, Aksum, Ethiopia; c Department of Pathology, Addis Ababa University College of Health Science, Addis Ababa, Ethiopia; d Department of Emergency and Critical Care Medicine, Aksum University College of Health Science, Aksum, Ethiopia; e Bahirdar University College of Health Science, School of Medicine, Bahirdar, Ethiopia

**Keywords:** case report, endometrial hyperplasia with atypia, Ethiopia, postmenopausal bleeding, vaginal leech infestation

## Abstract

**Background:**

Postmenopausal bleeding is a common gynecological complaint with multiple etiologies, ranging from benign to malignant conditions. Leech infestation of the female genital tract is a rare cause, particularly in rural regions with exposure to natural pooled water sources.

**Clinical presentation:**

We report a 50-year-old postmenopausal woman who presented with a 1-week history of vaginal bleeding. Ultrasound evaluation revealed a thickened endometrium (20 mm), and histopathology confirmed endometrial hyperplasia with atypia. She underwent total hysterectomy with bilateral salpingo-oophorectomy. Unexpectedly, persistent postoperative vaginal bleeding revealed a live leech attached to the vaginal vault, which was removed successfully with saline irrigation and forceps.

**Clinical discussion:**

The coexistence of both conditions poses a diagnostic challenge, as one condition can obscure the other. Leech attachment causes prolonged bleeding through its anticoagulant secretions, and its presence should be suspected in women from rural settings. A careful speculum examination remains crucial even when another etiology has been established.

**Conclusion:**

This case illustrates a rare coexistence of two distinct causes of vaginal bleeding: endometrial hyperplasia with atypia and vaginal leech infestation. In women from rural areas with exposure to contaminated water, clinicians should maintain a high index of suspicion for leech infestation even when another cause of bleeding is established.

## Introduction

Postmenopausal bleeding occurs in approximately 10% of women and accounts for nearly two-thirds of gynecologic clinic visits in this population^[^1^]^. Genitourinary atrophy is the most common cause, responsible for up to 60% of cases^[^2^]^. The other etiologies include both benign and malignant conditions of the genitourinary tract, such as endometrial hyperplasia (EH), which accounts for 15% of postmenopausal bleeding cases^[^1,3^]^.

EH is believed to result from prolonged exposure to unopposed estrogen, particularly in women with obesity, chronic anovulation, or those receiving menopausal estrogen replacement therapy^[^4^]^. Histologically, EH is characterized by diffuse glandular crowding, producing a gland-to-stroma ratio of 2:1, along with disorganized glandular architecture^[^5^]^.

HIGHLIGHTS

- Rare coexistence of endometrial hyperplasia with atypia and vaginal leech infestation in a postmenopausal woman.
- Persistent postoperative bleeding revealed an unexpected live leech attached to the vaginal vault.
- Highlights the diagnostic importance of speculum examination even after hysterectomy.
- Emphasizes vigilance for parasitic causes of bleeding in women from rural areas with water exposure.

Historically, EH was classified into four categories: simple and complex hyperplasia, each with or without atypia, based on the degree of glandular crowding and nuclear atypia^[^3,6^]^. However, this classification has since been simplified into two categories: hyperplasia without atypia and with atypia, as cytologic atypia has been recognized as the strongest predictor of progression to endometrial carcinoma rather than glandular complexity^[^7^]^.

Atypical EH carries a substantially higher risk of coexistence with or progression to endometrial carcinoma. In contrast, non-atypical hyperplasia carries only a 1–3% risk of progression over 10 years^[^8,9^]^. Recent data suggest that nearly half of women with EH are postmenopausal^[^10^]^. In this group, those with atypical hyperplasia are usually treated with a hysterectomy, given the elevated risk of malignancy and the absence of fertility concerns^[^3,11^]^.

Leech infestation of the female genital tract is a rare but noteworthy cause of postmenopausal bleeding, particularly in women living in rural areas. Leeches possess an oral sucker used for attachment and feeding and a caudal sucker that helps with movement^[^12^]^. Their size ranges from as small as 5 mm to as long as 45 cm, depending on their developmental stage and exposure to mammalian blood. It is primarily reported in rural settings, where women are exposed to stagnant or pooled river water, typically during bathing or swimming^[^13^]^.

Once attached to mucosal surfaces, they tend to remain for days or even weeks. Their initial attachment often goes unnoticed, as it is typically painless. Their saliva contains hirudin, a potent anticoagulant, which can lead to persistent bleeding at the site of attachment – sometimes lasting several hours or even days after removal^[^12^]^.

This case highlights the importance of maintaining a high index of suspicion for leech infestation when evaluating unexplained vaginal bleeding, especially in postmenopausal women from endemic or rural settings. Early identification is very important for appropriate treatment and to avoid unnecessary interventions and diagnostic delays. We report an unusual case of concurrent EH with atypia and vaginal leech infestation in a postmenopausal woman, a coexistence not previously documented in the literature. This case report is written according to the SCARE 2025 guidelines^[^14^]^.

## Case presentation

A 50-year-old para 6 woman, who has been amenorrheic for the past 4 years, presented to our gynecologic outpatient clinic with a primary complaint of vaginal bleeding of 1 week’s duration. The bleeding was initially described as intermittent, minimal in amount, and not associated with clots. She denied associated abdominal or lower back pain, pelvic pressure, or urinary symptoms. There was no history of trauma, recent sexual intercourse, or use of hormone replacement therapy.

She had no history of similar episodes of bleeding since menopause. Her menstrual history prior to menopause was unremarkable, with regular cycles of average flow. Obstetric history revealed six term spontaneous vaginal deliveries without complications. She had no history of pelvic or perineal surgeries.

She reported no history of chronic medical illness such as hypertension, diabetes mellitus, or thyroid disease, and she was not on any long-term medications. She denied the use of anticoagulants or traditional/herbal remedies. There was no family history of gynecologic or gastrointestinal malignancy.

The patient lives in a rural area with limited access to clean water and reported bathing regularly in nearby rivers. She did not recall any recent episodes of vaginal foreign body insertion or accidental trauma. Otherwise, she has no constitutional symptoms such as fever, night sweats, anorexia, or significant weight loss. She had not experienced a change in bowel habits or hematochezia. There was no prior history of cervical cancer screening or other gynecologic evaluation.

On presentation, the patient’s vital signs were within the normal range, and her body mass index was 27 kg/m^2^. General physical examination was unremarkable. The abdomen was flat and smooth, with no palpable mass, organomegaly, or signs of fluid collection. On genitourinary examination, the vaginal introitus appeared atrophic but intact, with no mass or ulceration. Sterile speculum examination revealed a narrowed but smooth vaginal canal and a grossly normal cervix with a small amount of blood but no active bleeding. Bimanual examination revealed no cervical motion tenderness, and the uterus was approximately normal in size, with no palpable adnexal mass.

Abdominopelvic ultrasound demonstrated a markedly thickened endometrium measuring 20 mm, homogeneous in echotexture, without obvious intrauterine or adnexal mass. In view of postmenopausal bleeding with thickened endometrium, the presumptive diagnosis of EH was made. The patient was counseled and underwent endometrial biopsy using a manual vacuum aspiration system under aseptic conditions. A sterile speculum examination performed at the time of biopsy revealed no vaginal foreign body, parasite, or active bleeding source. The specimen was then sent for histopathology evaluation.

One week later, before her scheduled biopsy result, she experienced progressive vaginal bleeding that persisted until her appointment visit. Histopathology revealed EH with atypia. Hematological evaluation showed a hemoglobin level of 10.9 g/dL; other hematologic and biochemical parameters, including liver and renal function tests, were normal. Given her postmenopausal status, histological findings, and ongoing bleeding, the patient was counseled for definitive surgical management with hysterectomy.

After informed written consent was obtained, she was prepared for total abdominal hysterectomy with bilateral salpingo-oophorectomy under spinal anesthesia. The abdomen was entered through a midline infraumbilical incision. Intraoperative findings were unremarkable: the uterus, adnexa, bowel, mesentery, and peritoneal surfaces showed no gross nodularity or suspicious lesions. Hysterectomy with bilateral salpingo-oophorectomy was performed in the standard manner. Hemostasis was secured, instrument counts were correct, and the abdomen was closed in layers. The intraoperative course was uneventful, and the patient was transferred to the recovery unit in stable condition. The hysterectomy specimen was subjected to histopathologic examination.

**Figure 1.**
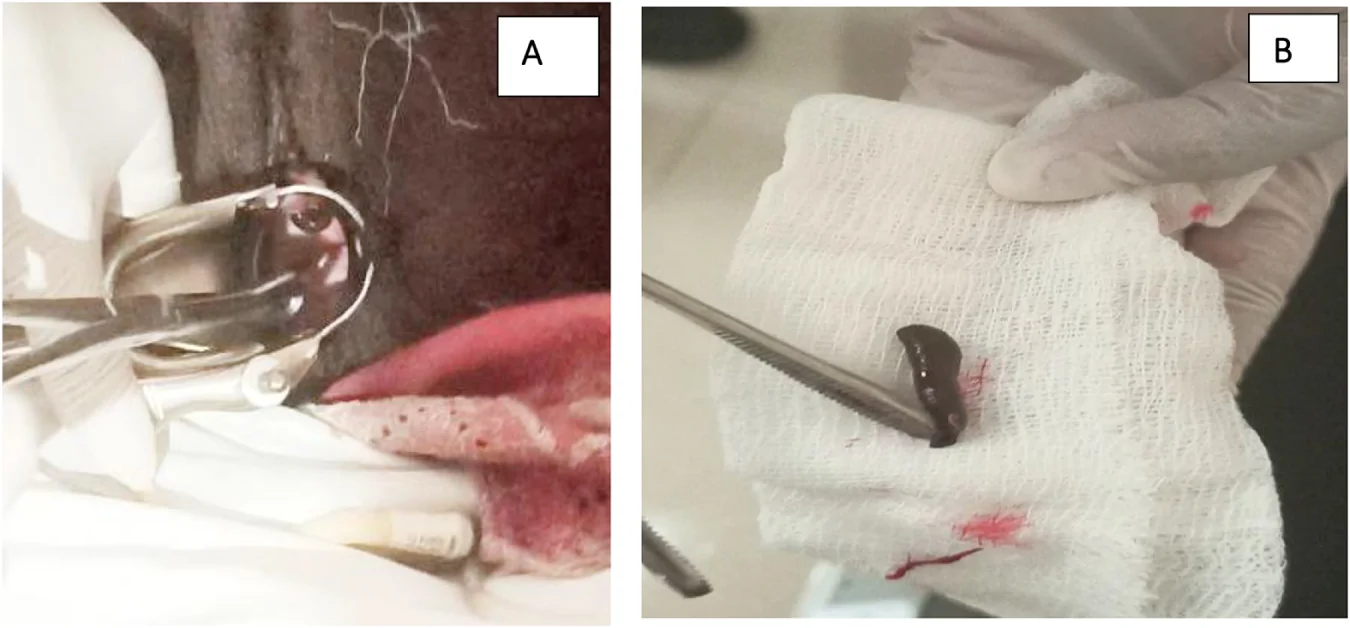
(A) Leech attached to the vaginal vault (arrow) during speculum examination. (B) Leech removed from the vaginal cavity, held with forceps over sterile gauze.

Unexpectedly, the patient continued to experience vaginal bleeding during the immediate postoperative period, initially attributed to retained blood within the vaginal canal. However, the bleeding persisted throughout the night, despite stable vital signs. Approximately 14 hours after surgery, a repeat sterile speculum examination of the vaginal vault was performed to exclude surgical site bleeding. To the surprise of the managing team, a live leech was found firmly attached to the right corner of the vaginal cuff (Fig. 1).

The vaginal canal was then irrigated with normal saline, prompting the leech to detach spontaneously. It was then grasped with sponge-holding forceps and removed intact. The mucosal bite site at the apex of the vaginal vault was oozing minimally, and a vaginal pack was applied to achieve compression hemostasis. Subsequently, the patient’s hemoglobin was rechecked and was 10 g/dL. She was observed closely for 3 days, during which her condition remained stable, and no further bleeding occurred. Oral iron supplementation was initiated for her mild anemia. She was discharged in good condition with follow-up instructions.

**Figure 2.**
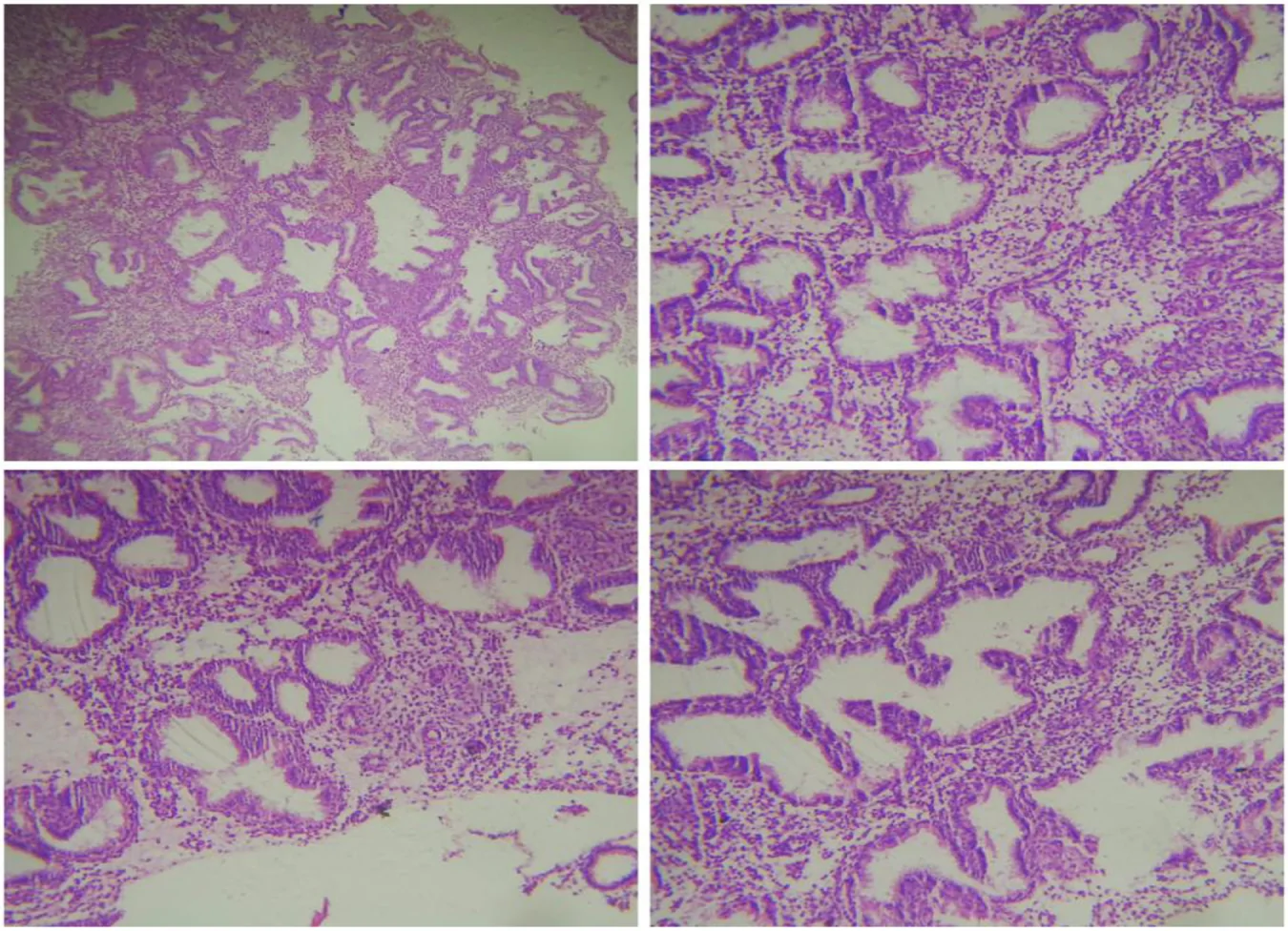
Hematoxylin and eosin microscopic images at different magnification displaying closely packed glands with gland to stroma ratio of 4:1, variation in gland size with cystic dilatation or irregular luminal contours, and associated with stromal breakdown. The glands are lined by columnar cells displaying loss of cellular polarity, hyperchromatic, and nuclear enlargement.

At her 1-week follow-up, she was clinically well, with no further bleeding, wound complications, or signs of infection. Histopathological examination of the hysterectomy specimen confirmed EH with atypia, consistent with the initial biopsy result. Hematoxylin and eosin-stained sections from the initial endometrial biopsy as well as the hysterectomy specimen showed closely packed glands with gland-to-stroma ratio of 4:1, variation in gland size with cystic dilatation or irregular luminal contours, and with associated stromal breakdown. The glands were lined by columnar cells displaying loss of cellular polarity, hyperchromatic nuclei with nuclear enlargement. No stromal invasion was noted (Fig. 2). Based on these histopathologic features, a diagnosis of EH with atypia was made. The patient and her family were counseled regarding the findings, and she expressed satisfaction with the resolution of her symptoms.

## Discussion

Postmenopausal vaginal bleeding is a serious gynecologic condition that requires thorough evaluation due to the risk of gynecologic malignancies. Although most cases are attributed to benign conditions such as genital tract atrophy, hormonal therapy, or EH, excluding malignancy remains the priority in clinical assessment^[^4^]^. The present case illustrates a rare diagnostic dilemma: the co-occurrence of a common cause of vaginal bleeding (EH) with a rare cause of vaginal bleeding (vaginal leech infestation).

In this patient, EH was diagnosed through ultrasound and histopathology after endometrial sampling. Considering her postmenopausal status and histologic finding, a total abdominal hysterectomy with bilateral salingo-oophorectomy was subsequently performed. Unexpectedly, vaginal bleeding continued on the night of the surgery. A repeat sterile speculum examination revealed an enlarged blood-sucking leech in the vaginal vault, an unusual and surprising finding in modern gynecologic practice.

This challenging scenario raised an important diagnostic question: was the leech infestation the primary cause of the bleeding or did it occur secondary to or concurrent with her underlying uterine pathology? The temporal sequence suggests that infestation likely occurred during the 2-week interval between histopathologic diagnosis and surgery. Given the patient’s residence in a rural area and regular bathing in stagnant river water, exposure risk was high^[^13^]^. This highlights the need for context-aware evaluation in women from endemic regions, where parasitic causes may coexist with more conventional pathologies. Importantly, no leech or vaginal abnormality was identified during the sterile speculum examination performed at the time of endometrial biopsy, supporting the likelihood that the infestation occurred after diagnostic sampling and prior to surgery.

EH is a well-established precursor of endometrial malignancy and is currently classified into two categories based on the presence or absence of cytologic atypia^[^3,8^]^. Earlier studies estimated a 1–3% progression rate to carcinoma for non-atypical EH^[^15^]^, but more recent research suggests a higher rate of up to 8.3%, possibly due to longer follow-up and inclusion of postmenopausal women^[^3^]^. Therefore, any degree of EH in postmenopausal women warrants comprehensive evaluation with transvaginal ultrasound and histologic sampling.

Leech infestation of the female genital tract is very rare, reported primarily in rural communities where exposure to stagnant water sources are common^[^16^]^. Leeches attach to mucosal surfaces using a caudal sucker and penetrate the mucosa using an oral sucker to feed on host blood. Their saliva contains a potent anticoagulant (hirudin), vasodilators, hyaluronidase, and calin, a platelet-aggregation inhibitor^[^17^]^. These substances collectively prevent coagulation, leading to prolonged bleeding even after the leech detaches. Because the bite is painless and occurs in warm, moist, hollow cavities, such as the vaginal canal, the infestation often remains unnoticed until bleeding becomes apparent^[^18,19^]^. In our case, the leech was detected after persistent postoperative vaginal bleeding leading to sterile speculum examination, which is vital especially in women from rural areas with potential exposure to stagnant and natural water bodies.

Potential complications of leech infestation extend beyond local bleeding. Persistent blood loss can cause hypovolemia, severe anemia, or circulatory collapse^[^20^]^. The anticoagulant effects of leech saliva exacerbate bleeding^[^12^]^, while the bite wound may serve as an entry point for secondary infections^[^21^]^. Additionally, leeches can act as mechanical vectors of pathogens, as they may retain bacteria, protozoa, and viruses, including hepatitis B and HIV in their gastrointestinal tract for extended periods. These pathogens can be transmitted even though they do not enter the salivary glands, especially if they are disturbed while feeding^[^22^]^. Forceful or incomplete removal can leave the jaws embedded in tissues, causing persistent bleeding and secondary infection^[^21^]^.

Management of vaginal leech infestation involves both supportive and definitive measures. Supportive care includes hemodynamic stabilization, blood transfusion, or iron therapy depending on the severity of anemia^[^20^]^. Definitive management requires careful removal of the leech to prevent residual jaw fragments^[^12^]^. Agents such as alcohol, salt, normal saline, or local anesthetic facilitate spontaneous detachment, allowing easy removal using forceps^[^20^]^.

In our case, normal saline was initially infused into the vaginal canal, prompting the leech to move. It was immediately grasped and removed using forceps. The mucosal bite site, located at the right corner of the vaginal vault, showed minimal but continued bleeding; a gauze pack was then inserted to provide compression and achieve hemostasis.

This case emphasizes that in regions where leech exposure is possible, clinicians should consider leech infestations as a potential cause of persistent vaginal bleeding even after major gynecologic surgery or when another etiology, such as EH, has been established. A simple speculum examination can be diagnostic and potentially lifesaving in such scenarios, particularly in resource-limited rural settings.

## Conclusion

The coexistence of EH with atypia and vaginal leech infestation is extremely rare. This case highlights the importance of maintaining broad diagnostic considerations in postmenopausal bleeding. Speculum examination should not be omitted, particularly in patients from rural areas with potential exposure to contaminated water sources. Early identification prevents unnecessary interventions and complications.

## Data Availability

All data generated or analyzed during this study are included in this published article.
